# Stewardship and COVID-19: The Preservation of Human Experience

**DOI:** 10.1177/1550190620981028

**Published:** 2021-09

**Authors:** Tory Schendel

**Affiliations:** 1 Evansville Museum of Arts, History & Science, Evansville, IN, USA

**Keywords:** COVID-19, museum, subject focus, case study, archives, collections assessment, digital collections, education, exhibition, metadata, provenance, records

## Abstract

During the current COVID-19 pandemic, museums, archives, and historical organizations are actively collecting material documenting these unusual times. The Evansville Museum of Arts, History & Science is one of the institutions active in this contemporaneous collecting. While this type of collecting follows in the footsteps of previous local efforts to document atypical times, I am no longer of the opinion this type of collecting—rapid response—should be doctrine or an expectation for 21st century curators. This article addresses the importance of democratizing trends in the museum field and allowing the curator, or person taking on the responsibility of collecting, to evaluate if one is truly capable of pursuing this type of collecting.

At the beginning of COVID, I reflected upon my role as a 21st-century curator and affirmed that part of my job is to “collect history in the making.”^1^ While I still perform this duty, I no longer of the opinion that rapid response^2^ should be doctrine or an expectation for 21st-century curators. In this next section, I hope to express the importance of democratizing trends in the museum field that put the curator, or person taking on the responsibility of collecting, in a vulnerable position. This article explores rapid response collecting initiatives such as fieldwork and participation in *A Journal of the Plague Year: An Archive of COVID-19* virtual repository, and evaluates if one is truly capable of pursuing this type of collecting.

Originally an advocate for rapid response collecting, I was a panelist for the presentation, *Collecting COVID-19*, for the American Alliance of Museums Virtual 2020 Annual Meeting and Expo. My section of the presentation focused on the rapid response collecting and methodology conducted at the Evansville Museum of Arts, History & Science. The presentation was conducted through the ZOOM application, which is accompanied by a chatbox feature where attendees can message panelists in real-time. As the presentation began, our moderator commented on how museum professionals can be affected by rapid response collecting and that professionals could feel trauma because this type of collecting is painful. Museum professionals collect a crisis in the “now”: the task can be emotionally and physically draining because professionals are collecting and documenting the human experience of a pandemic. Simultaneous to the ending of the moderator’s remark, a viewer commented on the chatbox: “You should be careful with the language around ‘painful collecting.’” The people who lived through those events are/were in pain, *not* the people collecting objects related to those events.”^3^ I regret not immediately interrupting the session to address this statement directly. Nonetheless, if I would have, I doubt my perspective about rapid response collecting as “doctrine” would have altered at that particular moment (Figure 1).

**Figure 1.**
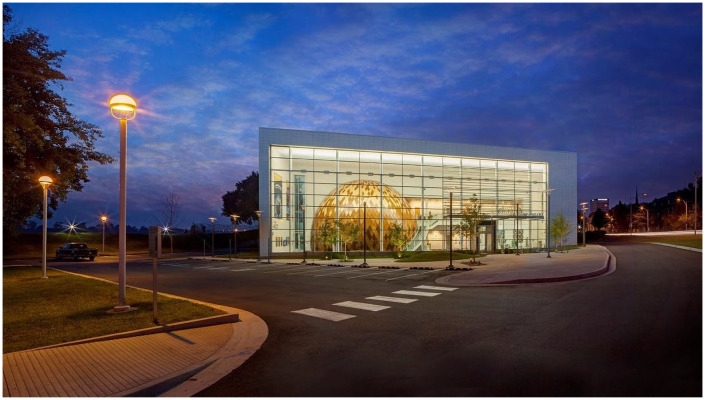
Image of the Evansville Museum of Arts, History & Science located in Evansville, Indiana. *Source*. For more information and image source, visit emuseum.org.

To further elaborate, I was given another opportunity to discuss rapid response collecting at the international conference *What Matters Now*, which was five weeks after the AAM conference. My presentation *Collecting in the “Now,”* was supposed to be a general overview of the COVID-19 collecting done at the Evansville Museum, similar to the presentation conducted at the American Alliance of Museums Virtual 2020 Annual Meeting. However, communication from the *What Matters Now* organizers to presenters, including me, indicated that the purpose of the conference was to offer a “free-style” presentation and offer meaningful content that focused on the humanistic aspects of rapid response collecting. The conference was broken down into “acts” and my presentation was included in “Act 1,” which read:

*Act 1: Coping with the Now* In the first, we will shed light on how people are responding to the present day as painful and stressful at times. How are we coping with the ongoing crises in the world? Our speakers and performers will shed a light on the situation and reactions in their respective communities and areas of expertise.^4^

In drawing up notes for my presentation, I wondered what the might audience gain from hearing an overview of our institutional rapid response collecting? Realistically, not much, if anything at all. Therefore, I decided to scrap my presentation in favor of using the *What Matters Now* platform to discuss the reality of rapid response collecting and the ramifications one can experience if one chooses to document this type of history. Heavily focusing on the comment made in the chatbox during the American Alliance of Museums presentation on June 4, 2020, I “free-styled” a presentation addressing the trauma one can feel and is allowed to feel while conducting “painful collecting.” Also, I used this platform to announce publicly that I no longer support the notion that rapid response collecting is a requirement or expectation of 21st-century curators or creative professionals.

Overwhelmingly, the participants were supportive of my newfound perspective. As conversations on this topic continued throughout the conference and afterward through emails and ZOOM calls, I concluded that rapid response collecting differs from traditional collecting practices because when one collects in the “now,” we are, at least in 2020, collecting human tragedy. Regarding COVID-19, this tragedy is continuing without an end in sight and this tragedy has a face and a response one can directly interact with. Due to this humanistic component, it is hard to boldly state the one collecting these stories do “*not*” feel pain. Reflect on therapists, first responders, service members, and those who deal with trauma daily. While they do not “collect” oral stories like curators, are they not empathetic to the person’s pain? Are they not allowed to feel emotional when someone shares their pain? If this were the case, why have peer-reviewed articles such as *The Emotional Impact of COVID-19: From Medical Staff to Common People; COVID-2019-Suicides: A Global Psychological Pandemic;* and *Suicide Mortality and Coronavirus Disease 2019*—*A Perfect Storm?*^5^ published by medical journals?

While it may seem remedial to some, museums not only unexpectedly closed because of the virus but according to the American Alliance of Museums, one-third of museums may not survive the fiscal impact and ramifications of COVID-19.^6^ With this in mind, how could one deem it acceptable for museum professionals to be excluded from the above research? Moreover, what ramifications are there for collections-building initiatives? How will the stories of today be represented in collections of the present and future? Will gaps in collections (due to items not being collected as rapid response measures), be tenable for museums? For museum professionals and their long-term well-being? What matter more: acquisition of collections or self-preservation of collections professionals?

With the closures of museums, as well as businesses, schools, and universities, almost every state in the union issued a “stay-at-home” order instructing residents to shelter in place and self-quarantine. Once Indiana declared the “state of emergency” order on March 23, 2020,^7^ the Evansville Museum of Arts, History & Science issued a memorandum stating that the museum would close and the staff was instructed to work at home. While I understand the importance and reasoning for the “stay-at-home” order, being removed from the museum’s permanent collection was one of the most difficult transitions. Nonetheless, from my graduate work and independent research, I reflected on academic materials that focused on curatorial practices in correlation to community crisis such as *History Museums and Social Cohesion: Building Identity: Bridging Communities and Addressing Difficult Issues; Rapid-Response Collecting after the Pulse Nightclub Massacre; K(NO)W Justice K(NO)W Peace: The Making of a Rapid-Response Community Exhibit* and the American Alliance of Museums 2017 presentation, *It Could Happen to You: Collecting in the Face of Tragedy*.^8^ Collectively, these sources note the importance of using one’s curatorial skillsets to help communities process tragedy because museums often become centers for reflection, memorization, and possibly mourning. To better understand how curatorial skillsets and museums can assist in the community comprehension, and possibly healing, process during or after a crisis, it is important to consider the perspective of Pam Schwartz, Chief Curator for the Orange County Regional History Center, who offered this testament in response to a shooting. In commenting on the June 12, 2016 shooting in Orlando, Florida, now referred to as the Pulse Nightclub Shooting, Schwartz stated:

. . .as a historian and curator, what am I supposed to be doing? Preserving the memory of this event for the education of those who are not living it. . .[Sunday,] I sat on the couch and wrote a five-page plan for how we need to collect this event. . .how are we going to preserve this for our community. On Monday, I handed this plan to my director. He just looked at me like ‘whoa too soon. No one is thinking about this but you’ well, this is the only thing I know how to do, it is my thing that I can do.^9^

Internalizing Schwartz’s remarks, the only thing I can do is create exhibitions and programming to interpret the human experience, which is something my community expects from me and my institution. This expectation is laid bare in community engagement surveys and data collection over the past several years.

## Rapid Response Collecting Conducted at the Evansville Museum of Arts, History & Science and Personal Reflection

**Figure 2.**
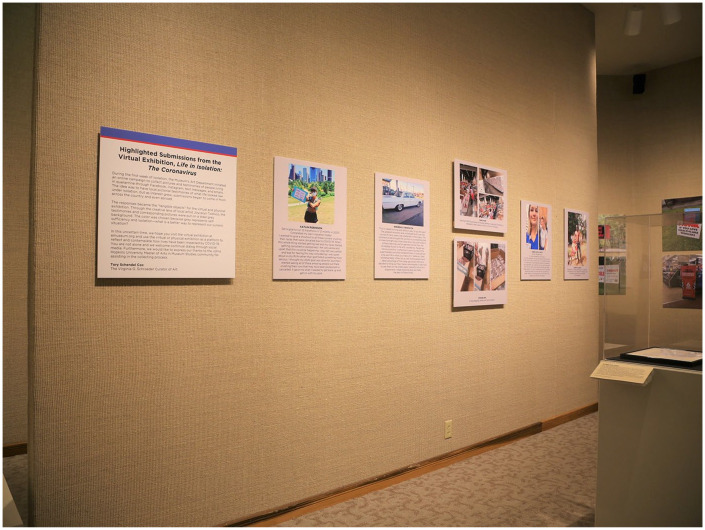
Image of the Main Gallery in the Evansville Museum of Arts, History & Science display of highlighted submissions from the virtual exhibition “Life in Isolation: The Coronavirus.” By printing the entries on foam core board, the virtual crowdsourced materials become tangible objects for a physical exhibition. *Source*. For more information and image source, visit emuseum.org.

During the first week of lockdown, I started an online campaign to collect pictures and testimonies of people living in quarantine through Facebook, Instagram, and emails. As interest grew, I included phone calls and text messages for accessibility purposes. This campaign was charged with the idea for local pictorial testimonies to be made available. As this exhibition grew, people across the country as well as internationally submitted content. In conjunction with the virtual exhibition, I started a mini-series called “Cultural Insights: Interviews in the Creative Sector.” It came together organically. Originally, it was supposed to be a “feel good” series where creatives talked about their work. Nonetheless, how can one talk about their work without mentioning COVID-19? From there, it turned into a platform where creatives discuss who they are and how creatives triumph through the hardship of COVID-19. The virtual exhibition, *Life in Isolation: The Coronavirus*, and the video program, *Cultural Insights: Interviews in the Creative Sector*, sought to address community questions: (1) What does life in isolation this mean, (2) What does life in isolation look like, and (3) How is our community coping? Since this pandemic is ongoing, we are continually evaluating these questions (Figure 2).

From the collecting process and reflecting on the stories from the newly unemployed to high-risk individuals and even some people fortunate enough to feel relatively unaffected: when one reaches out and attempts to collect the “actual” human experience, it became increasingly hard. The more friends, colleagues, and community members I interacted with, the more I grew to understand the impact of COVID-19. From the beginning to the present day, I have personally known twenty-four people who contracted COVID-19 and twelve who have passed because of the virus. Because I unknowingly reached out, individuals entrusted me with their experience. Whether it is first or second hand, trying to comfort someone who is experiencing the brunt of the virus is one of the most terrifying practices and above my skillsets. In the article, *Rapid-Response Collecting after the Pulse Nightclub Massacre*, some professionals shared their experience with collecting artifacts from the Pulse Nightclub Massacre. From the staff and volunteer reflections, I am more empathetic to their rapid response initiative because I can better understand how rapid response “collecti[ng] has been an exhausting and at times emotional assignment.”^10^

**Figure 3.**
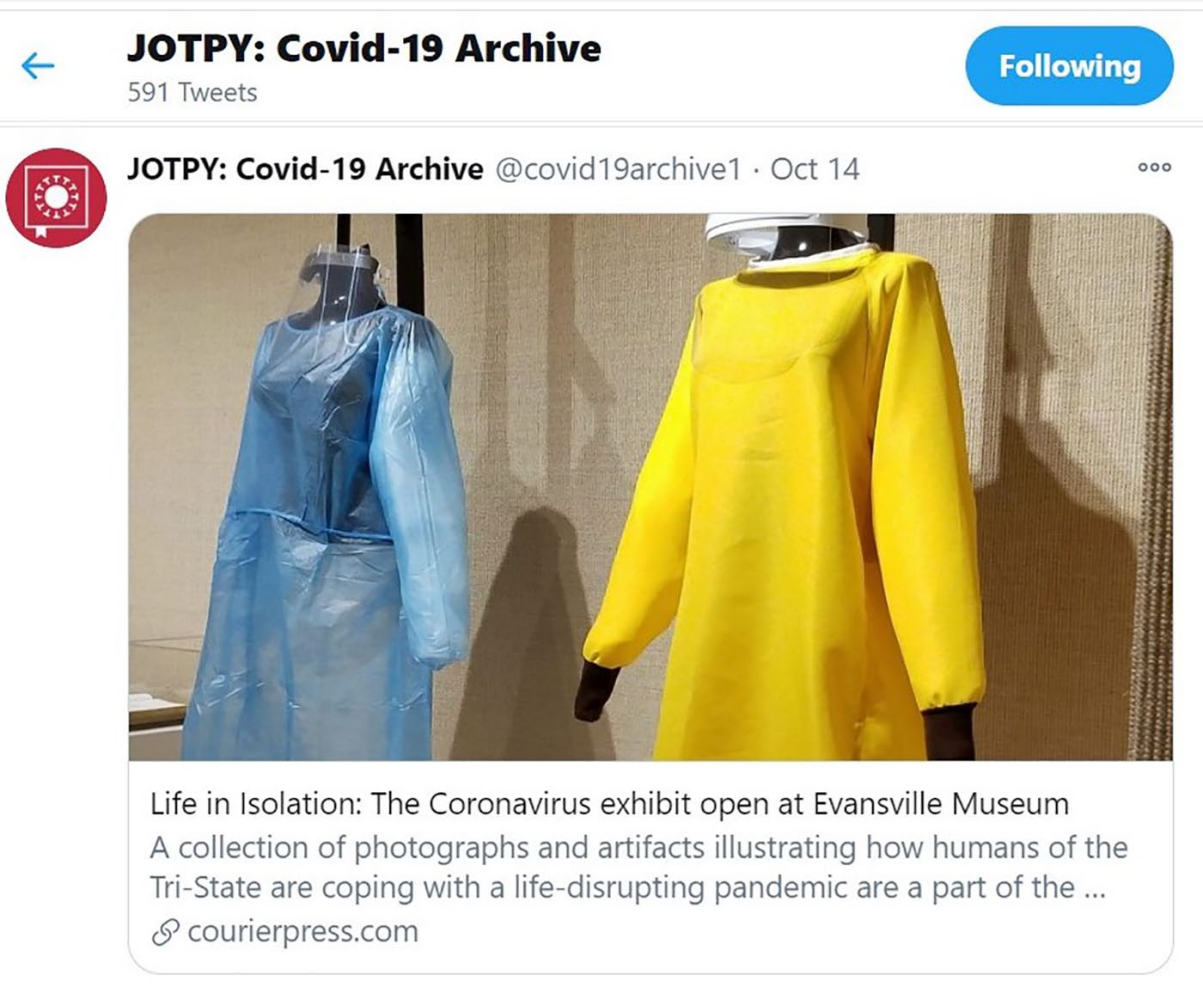
By sharing content, JOPY and the Evansville Museum is able to reach broader audiences and can mutually use the virtual repository to strengthen our understanding of the human experience during this pandemic. *Source*. Image source: https://twitter.com/covid19archive1.

Although I would never close my “virtual door,” the overwhelming experience of collecting human experience has made me reevaluate the notion that rapid response collecting should be a standard 21st-century curatorial practice. No museum professional should be shamed, slandered, or told how they should or should not feel, or if they should or should not perform rapid response collecting, especially from those in the museum community. No museum professional should feel embarrassed for exhibiting emotions while performing their method of work and if the person does not or cannot become emotionally invested in collecting the human experience, I raise two questions: (1) is the experience worth documenting at an institutional level? and (2) are you the best candidate to carry out this type of collecting? While these questions have more than one response—because the answer will greatly depend on one’s institution, mission statement, staff, and Collections Management Policy—it is important that every museum evaluate the importance of collecting in a crisis, or about 2020, the COVID-19 pandemic. Regardless of your institution’s decision, keep in mind, if one conducting rapid response collecting is unable to become emotionally invested or is uninterested in rapid response collecting, how can the institution expect their audience to support the documentation process and share personal testimonies if there is no underlining trust with the donor’s feelings? To this question, we are fortunate to live in the 21st-century and have solutions to this dilemma (Figure 3).

## A Journal of the Plague Year: An Archive of COVID-19 (JOTPY)

While I am no longer of the opinion that curators should be expected to undertake rapid response collecting, I do believe collecting the history and human experience of a crisis is important. Those who are willing to perform this type of work should be able to do so and be supported. Specifically, I would like to highlight the repository, *A Journal of the Plague Year: An Archive of COVID-19 (JOTPY)*. This online inclusive database of community crowdsourced collected materials allows for people across the country and abroad to submit and share their COVID-19 experience. Such a crowdsourcing initiative is important because the repository enables museum and academic professionals to reflect and study the human experience of COVID-19 without having to collect themselves. As a contributor to the repository, I am not discouraging those who feel compelled to document the pandemic but am hoping to advocate other alternatives and democratize the decision-making process of rapid response collecting. Nonetheless, being affiliated with the *JOPY* repository at an institutional level has been a rewarding experience because our community collected materials and stories are available for reflection and memorialization on an international level. By having a global presence, we can serve a broader community and contribute to a platform that centralizes COVID-19 collected materials at no cost to the observer. By centralizing COVID-19 materials, information becomes easily accessible and allows *JOPY* to create a meaningful and equaling impactful digital experience for future viewers.

## Conclusion

Professionals are human. And as noted above, COVID-19 has a face and persona. Therefore, I hope professionals will evaluate their skillsets and capabilities while preserving and protecting their own mental health before they choose to conduct rapid response collecting. As this pandemic continues, we are in this together and need to persevere as one unified community.

## Footnotes

1. Tory Luel Schendel Cox, “Collecting and Unifying Under a Pandemic: COVID-19,” *Informal Learning Review*, 2 (2020): 31–2. https://informallearning.com/storage/issues/ILR-Special-Issue-2020-2.pdf.

2. Rapid Response Collecting is also referred to as Crisis Collecting. The words may be capitalized and/or hyphenated, or not.

3. Collecting COVID-19, “AAM Virtual Conference: Collecting COVID-19,” 4 June 2020, available at: https://aamvirtual.elevate.commpartners.com/products/collecting-covid-19.

4. Program. “What Matters Now,” 10 July 2020, available at: http://www.whatmattersnow.live/program

5. Nicola Montemurro, “The Emotional Impact of COVID-19: From Medical Staff to Common People,” *Brain, Behavior, and Immunity*, 87 (2020): 23–4. https://www.ncbi.nlm.nih.gov/pmc/articles/PMC7138159/; Vikram Thakur and Anu Jain, “COVID 2019-Suicides: A Global Psychological Pandemic,” *Brain, Behavior, and Immunity*, 88 (2020): 952–53. https://pubmed.ncbi.nlm.nih.gov/32335196/. ; Mark A. Reger, Thomas E. Joiner, and Ian H. Stanley. “Suicide Mortality and Coronavirus Disease 2019-A Perfect Storm?” *JAMA Psychiatry*, 10 April 2020, available at: https://jamanetwork.com/journals/jamapsychiatry/fullarticle/2764584

6. American Alliance of Museums, “United States May Lose One-Third of All Museums, New Survey Shows,” 22 July 2020, https://www.aam-us.org/2020/07/22/united-states-may-lose-one-third-of-all-museums-new-survey-shows/

7. Eric Holcomb, “State of Indiana: Executive Department Indianapolis,” 20 April 2020, available at: https://www.in.gov/gov/files/Executive%20Order%2020-22%20Extension%20of%20Stay%20at%20Home.pdf

8. Tracy Jean Rosenberg, “History Museums and Social Cohesion: Building Identity, Bridging Communities, and Addressing Difficult Issues,” *Peabody Journal of Education*, 86, no. 2 (2020): 115–28. https://www.tandfonline.com/doi/abs/10.1080/0161956X.2011.561171; Pam Schwartz, Whitney Broadaway, Emilie S. Arnold, Adam M. Ware, and Jessica Domingo, “Rapid-Response Collecting after the Pulse Nightclub Massacre,” University of California Press, 1 February 2018, available at: https://online.ucpress.edu/tph/article/40/1/105/90936/Rapid-Response-Collecting-after-the-Pulse; Brenda Tindal, “K(NO)W Justice K(NO)W PeaceThe Making of a Rapid-Response Community Exhibit,” University of California Press, 1 February 2018, available at: https://online.ucpress.edu/tph/article/40/1/87/90985/K-NO-W-Justice-K-NO-W-PeaceThe-Making-of-a-Rapid; Adam Ware, Amy Weinstein, George McDaniel, Pam Schwartz, Tamara Kennelly, “It Could Happen to You: Collecting in the Face of Tragedy,” May 2017, available at: www.aam.shop.webcast.guru/?download=3991-it-could-happen-to-you-collecting-in-the-face-of-tragedy

9. Ibid.

10. Schwartz, Broadaway, Arnold, Ware, and Domingo, 2018, 111.
